# The U2AF65/circNCAPG/RREB1 feedback loop promotes malignant phenotypes of glioma stem cells through activating the TGF-β pathway

**DOI:** 10.1038/s41419-023-05556-y

**Published:** 2023-01-13

**Authors:** Hao Li, Yang Jiang, Jinpeng Hu, Jinkun Xu, Lian Chen, Guoqing Zhang, Junshuang Zhao, Shengliang Zong, Zhengting Guo, Xinqiao Li, Xiang Zhao, Zhitao Jing

**Affiliations:** 1 Department of Neurosurgery, The First Hospital of China Medical University, No. 155 North Nanjing Street, Shenyang, 110001 China; 2 Department of Neurosurgery, Shanghai Tenth People’s Hospital, Tongji University School of Medicine, Shanghai, 200072 China

**Keywords:** Cancer stem cells, Oncogenes

## Abstract

Glioma is the most aggressive and common malignant neoplasms in human brain tumors. Numerous studies have showed that glioma stem cells (GSCs)drive the malignant progression of gliomas. Recent studies have revealed that circRNAs can maintain stemness and promote malignant progression of glioma stem cells. We used bioinformatics analysis to identify circRNAs and potential RNA-binding proteins (RBPs) in glioma. qRT-PCR, western blotting, RNA FISH, RNA pull-down, RNA immunoprecipitation assay, ChIP, immunohistochemistry, and immunofluorescence methods were used to quantified the expression of circNCAPG, U2AF65, RREB1 and TGF-β1, and the underlying mechanisms between them. MTS, EDU, neurosphere formation, limiting dilution neurosphere formation and transwell assays examined the proliferation and invasive capability of GSCs, respectively. We identified a novel circRNA named circNCAPG was overexpressed and indicated the poor prognosis in glioma patients. Upregulating circNCAPG promoted the malignant progression of GSCs. RNA binding protein U2AF65 could stabilize circNCAPG by direct binding. Mechanically, circNCAPG interacted with and stabilized RREB1, as well as stimulated RREB1 nuclear translocation to activate TGF-β1 signaling pathway. Furthermore, RREB1 transcriptionally upregulated U2AF65 expression to improve the stability of circNCAPG in GSCs, which established a feedback loop involving U2AF65, circNCAPG and RREB1. Since circRNA is more stable than mRNA and can execute its function continuously, targeting circNCAPG in glioma may be a novel promising therapeutic.

## Introduction

Glioma is the most aggressive and most prevalent malignant neoplasms of the central nervous system [1]. Despite surgery, radiotherapy and chemotherapy, glioma patients commonly recur with five-year survival rates improving from only 4% to 7% [2, 3]. Glioma contains a class of stem cell-like tumor cells named glioma stem cells (GSCs)that are considered to be tumor initiating cells, inducing the malignant phenotypes of gliomas in differentiation, proliferation, invasion, drug resistance and recurrence [4–6]. Therefore, identifying the molecular mechanisms in maintaining the stemness of GSCs and promoting the malignant progression, and discovering new molecular targets is crucial for glioma patients.

Circular RNAs (circRNAs) are a type of head-to-tail covalently closed noncoding RNA formation from the back splicing of precursor mRNAs [7]. Due to the circular structure, circRNAs are extremely stable compared to the linear RNAs and perform a variety of biological functions [8]. Numerous studies have reported that circRNA plays a vital role in regulating cancer progression, suggesting that circRNAs could serve as diagnostic and therapeutic targets [9–12]. A large amount of literature reports that various circRNAs act as miRNA sponges to perform their biological functions in cancer. In glioma, circATP5B competitively binds to miR-185-5p to increase HOXB5 expression, which promotes tumor progression [13]. CircMMP9 promotes tumorigenesis of glioblastoma via sponging to miR-124 and increasing the expression of CDK4 and AURKA [14]. However, recent studies have reported that circRNAs can directly bind to different proteins, regulating the function of related proteins and even being translated in glioma [15, 16]. Further understanding of the function and mechanisms of circRNAs in regulating GSCs progression may provide insight into tumorigenesis and progression of glioma.

Ras responsive element binding protein 1(RREB1) is a zinc finger transcription factor and regulates gene expression by binding RAS responsive elements (RREs) in the gene promoters [17]. It is an evolutionarily conserved protein found in a number of tissues and organs that may both act as a transcriptional repressor and activator, and has recently been shown to associated with cancer progression regulation [17, 18]. CircITGA7 inhibits colorectal cancer proliferation by upregulating ITGA7 expression via inhibition of RREB1 [19]. RREB1 is downregulated in PDAC and its downstream target gene ZIP3 is subsequently downregulated to prevent pancreatic adenocarcinoma cells death from cytotoxicity of zinc [20]. However, the biological functions and mechanism of RREB1 in the pathogenesis and progression of gliomas are uncertain.

In this study, we identified hsa_circ_0069280, derived from NCAPG, as an oncogene that was overexpressed in glioma, promoting GSCs progression. Moreover, U2AF65 is the RNA-binding protein that interacts with and regulates the expression of circNCAPG via stability according to bioinformatics and molecular experiments. Functionally, circNCAPG promotes glioma progression by stabilizing and promoting nuclear translocation of the RREB1 protein, allowing the TGF-β1 signaling pathway to be activated. Further, RREB1 could transcriptionally upregulate NESTIN expression to maintain stemness and U2AF65 expression to promote the stability of circNCAPG in GSCs. Our findings support the existence of a novel feedback loop U2AF65/circNCAPG/RREB1 that promotes malignant progression in GSCs and could be a novel therapeutic target for glioma patients.

## Materials and methods

### Ethics

The experimental protocol in this study was approved by the Ethics Committee of The First Hospital of China Medical University (AF-SOP-07-1.1-01). Clinical samples from a total of 70 glioma patients were acquired from January 2007 to January 2012 at the First Affiliated Hospital of China Medical University. These included 20 samples of WHO grade II, 25 samples of WHO grade III, and 25 samples of WHO grade IV glioma. In addition, adjacent nontumorous brain tissues (NBT) were collected from 10 of these patients as negative controls. Patient clinical information are available in Table 1.

**Table 1:** Relationship of circNCAPG expression to clinical features of glioma patients.

Clinical features		Samples (*n* = 70)	CircNCAPG expression^a^		*P*-value
			Low (*n* = 35)	High (*n* = 35)	
Sex	Male	36	17	19	*P* = 0.371
	Female	34	18	16	
Age	≤50	24	16	8	*P* = 0.284
	>50	46	19	27	
WHO grade	II	20	18	2	***P*** < **0.001**
	III	25	13	12	
	IV	25	4	21	
IDH status	Wild	33	9	24	***P*** = **0.015**
	Mutant	37	26	11	
1p/19q status	Codeletion	36	21	15	***P*** = **0.029**
	Non-codeletion	34	14	20	
H3F3A status	Wild	41	28	13	***P*** = **0.007**
	Mutant	29	7	22	
MGMT status	Methylation	42	27	15	***P*** = **0.011**
	Unmethylation	28	8	20	
Survival time (months)	≤30	39	13	26	
	>30	31	22	9	

^a^CircNCAPG expression was detected by qRT-PCR and ranked from low to high. The high expression of circNCAPG was defined as the expression level higher than the median expression level of circNCAPG. Bold values identify statistical significance (*P* < 0.05).

### Data source and Bioinformatics analysis

All datasets were obtained from public databases. R package limma was used to analyze differential expression gene [21]. GSE109569 microarray dataset containing 3 paired glioma tissues was used to identify differential expression circRNAs in glioma. mRNA sequencing data from glioma patients were obtained from the Cancer Genome Atlas (TCGA) and the Chinese Glioma Genome Atlas (CGGA) mRNAseq_325 datasets. The TCGA and CGGA datasets were divided into two groups based on the median RREB1 expression and identified the enrichment pathways in the high RREB1 expression group using R package clusterProfiler [22]. ssGSEA was used to assess the TGF-β1 pathway enrichment score [23]. All websites are listed in Supplementary Table 1.

### Cell culture and GSCs isolation

Human glioma cell lines U87, U251 were purchased from the Chinese Academy of Sciences cell bank (Shanghai, China). The cell line LN229 obtained from the American Type Culture Collection (Manassas, VA). Human glioma cell lines U87, U251, and LN229 were cultured in Dulbecco’s modified Eagle’s medium (DMEM, Gibco) with 10% fetal bovine serum (Gibco). When the cells were grown to a density of about 80%, they were dissociated into single cells using 0.25% trypsin (Gibco). After washing by with phosphate-buffered saline (PBS, Gibco) for 3 times, they were cultured in the DMEM/F12 (Gibco) medium supplemented with 2% B27 (Gibco), recombinant human basic fibroblast growth factor (rh-bFGF, 20 ng/ml, Gibco), and epidermal growth factor (rhEGF, 20 ng/ml, Gibco) at 37 °C with 5% CO2. The serum-free medium with growth factor was replaced every 3 days. Patient-derived GSC cells (GSC23, GSC27, GSC35) were extracted and cultured within serum-free DMEM/F12 containing 2% B27, 20 ng/mL rh-bFGF, and rh-EGF according to previous protocol [13, 24]. All cells had passed mycoplasma and the short tandem repeat (STR) DNA profiling tests.

### Lentivirus and transfection

The negative controls and the lentivirus vectors used for the overexpression of circNCAPG, U2AF65, and RREB1, as well as the RNAi mediated silencing of circNCAPG, U2AF65 and RREB1, were all produced by Gene-Chem (GV358, Shanghai, China). The siRNA sequences are performed in Supplementary Table 2.

### qRT-PCR and RNase R treatment

We performed real-time quantitative reverse transcription PCR as previously described [13]. Briefly, total RNA from glioma tissues as well as GSC cell lines was extracted using the RNA-easy Isolation Reagent (Vazyme). Reverse transcription (RT) was performed with the use of Prime Script RT Master Mix reagent kit (TaKaRa). qPCR was performed using the SYBR Green Master Mix (TaKaRa) with PCR LightCycler480 (Roche Diagnostics, Basel, Switzerland). In addition, we also used RNase R (Epicenter Technologies, Madison, USA) to detect the presence of circNCAPG and to remove the influence of linear NCAPG RNA. Gapdh was set as internal reference. Primers used in this study are listed in Supplementary Table 2.

### Western blotting

RIPA lysis with PMSF and 8 M Urea were used to extract protein of GSC cell lines. The nuclear and cytoplasmic proteins were extracted separately using a NE-PER™ Nuclear and Cytoplasmic Extraction Reagents (Thermo Fisher Scientific). The protein concentration was detected by BCA method and 15-30ug protein was loaded per well in and separated by SDS-PAGE. The polyvinylidene difluoride (PVDF) membranes with 0.22um pore size were indicated primary antibodies against U2AF65 (1:1000; Abcam), RREB1 (1:1000; Abcam), NESTIN (1:1000; Abcam), OCT4(1:1000; Abcam),SOX2 (1:1000; Abcam), Nanog (1:1000; Abcam), CD133 (1:1000; Abcam), TGF-B1 (1:1000;Cell Signaling Technology), SMAD2 (1:1000; Cell Signaling Technology), pSMAD2 (1:1000; Cell Signaling Technology), SMAD3(1:1000; Cell Signaling Technology), pSMAD3(1:1000; Cell Signaling Technology), lamin B1(1:1000; Proteintech), β-actin(1:1000; Proteintech). Following 1 h incubation with secondary antibody (ProteinTech), the signals were examined by chemiluminescence Femto-sig ECL kit (Tanon, Shanghai, China).

### RNA Fluorescence in situ hybridization (RNA FISH)

Cy3-labeled circNCAPG probes were designed and produced by Sangon Biotech (Shanghai, China). GSC cells from patients and U87 cell line were cultured on Confocal culture dishes, fixed with 4% paraformaldehyde for 25 min and permeated with 0.5% Triton X-100 for 10 min. The cells were incubated with hybridization solution containing 1 ng/uL circNCAPG probe overnight at 37 °C. The nuclei were stained with DAPI after rinsing the confocal culture dishes three times with hybridization buffer. Images were captured with the use of a confocal microscope (Nikon C1).

### Cell viability assay

A density of 1000 GSCs /well were cultured in 96-well plates for 5 days. The cell viability was then determined using the CellTiter 96® Aqueous Non-Radioactive Cell Proliferation Assay Kit (Promega, Madison, WI, USA) as previously described [13].

### EdU assay

The EdU assay kit (Beyotime, Biotechnology) was used to detect cell proliferation according to the manufacturer’s protocol. In brief, a density of 1 × 10^5^ GSCs/well were seeded into 24-well plates and cultured 24 h. Following 10 µm EdU reagent was added and incubated for 2 h, GSCs were fixed with 4% paraformaldehyde for 15 min and permeated with 0.3% tritonX-100 for 10 min, and were counterstained with DAPI. The positive cells were identified using a fluorescence microscope (Olympus).

### Transwell invasion assay

As previously described [13], 1 × 10^5^ GSC cells were suspended in 200 uL DMEM medium with 5% FBS and then plated into the upper chamber (Corning, NY, USA) with a Matrigel-coated filter. The medium containing 20% FBS was added to the lower chamber. Then the cells were cultured for 24 h. Following fixed with 4% paraformaldehyde for 20 min, the chambers were incubated with 0.1% crystal violet (Beijing Solarbio Science & Technology Co., Ltd.). Images of invasive cells were obtained using a light microscope (Olympus) and counted with ImageJ software.

### Enzyme-linked immunosorbent assay

ELISA was performed as previous description [13]. Measurement of the TGF-β1 concentration in media supernatants derived from GSCs was performed using a TGF-β1 Quantikine ELISA kit (R&D Systems, Minneapolis, MN, USA).

### Neurosphere formation

The neurosphere formation assay was performed as previous description [24]. A total of 200 GSCs/well were cultured in 24-well plates for 7 days. Images were acquired by using a microscope (Olympus), and the neurosphere was measured using ImageJ software.

### Limiting dilution neurosphere formation assays

The Limiting dilution neurosphere formation assays were performed as previous description [25]. Briefly, GSCs from dissociated neurospheres were plated into 96-well plates at a density of 1, 10, 20, or 30 cells/well and cultured for 7 days. The neurospheres larger than 50 mm in diameter were counted and R package statmod was used to analyze neurosphere synthesis efficiency [26].

### Luciferase reporter assay

Luciferase reporter assays were performed as previously described [27, 28]. The luciferase reporter plasmid (wild or mutant type of U2AF65 and NESTIN) were constructed by Genechem (Shanghai, China). The mutant type of U2AF65 and NESTIN contained two mutant sites (#1 and #2) according to the prediction of Jaspar database. 5 × 10^4^ GSC cells/well were plated into 48-well plates and cultured to 70% confluence, and the luciferase reporter plasmids were then co-transfected into GSC for 48 h. Relative luciferase activity was determined using the Dual-Luciferase Reporter Assay System (Promega).

### Chromatin immunoprecipitation assay (ChIP)

By using the ChIP Assay Kit (Beyotime Biotechnology), the chromatin complexes were immunoprecipitated with different antibodies respectively, according to manufacturer’s protocol (U2AF65, NESTIN, IgG antibodies). qPCR was used to determined purified DNA from ChIP assay.

### RNA immunoprecipitation assay (RIP)

RNA-Binding Protein Immunoprecipitation Kit (Millipore) was used to perform RIP assay according to manufacturer’s protocol. Approximately 1 × 10^7^ GSC cells under different conditions were collected respectively and then lysed in 100 ul of prepared RIP lysis buffer. The cell lysates were incubated with magnetic beads conjugated with normal rabbit IgG or U2AF65 antibodies at 4 °C overnight. Subsequently, the immunoprecipitated RNAs were isolated and examined by qRT-PCR.

### RNA pull-down assay

RNA pull-down assays were performed to confirmed the interaction between circNCAPG and U2AF65 by using the Pierce Magnetic RNA Protein pull-down Kit (Thermo Fisher Scientific) according to the manufacturer’s instructions. After labeling purified RNA with biotinylated RNA probes, positive control (input), negative control (antisense RNA) and biotinylated RNA were mixed and then incubated with GSC protein lysates. The probe-magnetic bead complexes were prepared by adding magnetic beads into the previous RNA-protein mixture and subsequently washing, boiling the complexes. RNA binding proteins were identified by western blotting.

### Liquid chromatography-mass spectrometry (LC–MS/MS)

Mass spectrometry assays were performed according to the manufacturer’s protocol. Briefly, after pull-down with biotin-labeled circNCAPG probe, the protein digested with protease into peptide. The enzymatically cleaved peptide samples were dissolved in Nano-LC mobile phase A (0.1% formic acid) and separated by ultra-high performance liquid chromatography system Easy-nLC 2000 (ThermoFisher, USA). Peptides were analyzed by mass spectrometry using a Q Exactive system (ThermoFisher, USA) combined with a Nano Flex ion source (ThermoFisher, USA) with nanoliter spray. The LC-MS/MS raw data was processed and retrieved using PEAKS Studio 8.5 (Bioinformatics Solutions Inc. Waterloo, Canada) software, and the database was the Homo sapiens protein database downloaded from Uniprot. The mass error was set to 10 ppm for precursor ions and 0.05 Da for fragment ions.

### RNA stability evaluation

Total RNA of GSCs in different conditions was isolated and treated with Actinomycin D (ActD; NobleRyder, China) at different time points. qRT-PCR was used to determined circNCAPG expression and the time point 50% reduction in CircNCAPG expression was confirmed.

### Protein stability evaluation

The treatment of MG-132 and cyclohexylamine (CHX) with GSCs was used to calculate the stability of the proteins. After GSC27 and GSC23 were transfection with circNCAPG knockdown or circNCAPG overexpression at 0 h. The cells were treated with MG-132(50 µm, Sigma-Aldrich) for 6 h or CHX (100 ng/ml, Sigma-Aldrich) for 0, 6, 12, 24, 36 h, the total proteins were extracted and detected by western blotting.

### Immunofluorescence

A density of 4 × 10^4^ GSC cells were plated into confocal culture dish and cultured for 24 h. Following fixing with 4% paraformaldehyde for 10 min, cells were permeated with 0.5% Triton X-100 for 20 min, and then blocked for 30 min with 5% bovine serum albumin (BSA). The primary antibody RREB1(1:50; Abacam) was then added and incubated at 4 °C overnight. After washed three times with PBS the next day, the cells were subsequently incubated with the secondary antibodies (ProteinTech) for 1 h at 37 ˚C. The nuclei were counterstained with DAPI and imaged with the use of a laser scanning confocal microscope (Olympus).

### Immunohistochemistry (IHC)

We performed immunohistochemistry assay using UltraSensitive TM SP IHC Kit (MaxVision Biotechnology, Fuzhou, China) according to manufacturer’s protocol. In brief, paraffin-embedded sections were processed using the IHC Kit and subsequently incubated with primary antibodies against Ki-67 (1:1000; ProteinTech), TGF-B1(1:100; Abcam), RREB1(1:100; Abcam), U2AF65(1:100; Abcam) at 4 °C overnight. The sections were incubated with a secondary antibody from the IHC Kit for 30 min at 37 ˚C and then imaged under a light microscope (Olympus).

### Xenograft tumor model

Xenograft tumor model were performed as previously described [27]. Six-week-old female BALB/c nude mice (Beijing Vital River Laboratory Animal Technology, Beijing, China) were raised at the Laboratory Animal Center of China Medical University. We divided the mice into seven groups with five mice in each group (Control, circNCAPG−OE, circNCAPG−KD1, RREB1 − KD1, circNCAPG−OE + RREB1 − KD1, U2AF65 − OE, U2AF65 − OE + circNCAPG−KD1). Using a stereotaxic device, 5 × 10^4^ cells of GSCs were injected orthotopically into the mouse brains at 2 mm lateral and 2 mm anterior to the bregma. Mice were examined daily and executed at the point of death. The tumor volume was measured according to the following formula: V = (D × d^2^)/2. All animal experiments were performed in accordance with the Animal Care Committee of China Medical University (KT2020091).

### Statistical analysis

All results from at least three times were analyzed by using the chi-square test, two-tailed Student’s *t*-test, or one-way analysis of variance. The correlation between the two groups analyzed using Pearson’s correlation analysis. Log-rank test and Kaplan–Meier analysis were used to assess the survival difference. GraphPad Prism 8.0.2 (GraphPad Software Inc, San Diego, C.A, USA) or R software 4.0.5 were used for all analyses and *P* < 0.05 were deemed significant. The data were graphed as mean ± SD.

## Results

### CircNCAPG is up-regulated in glioma tissues and indicates poor prognosis in glioma patients

**Fig. 1:**
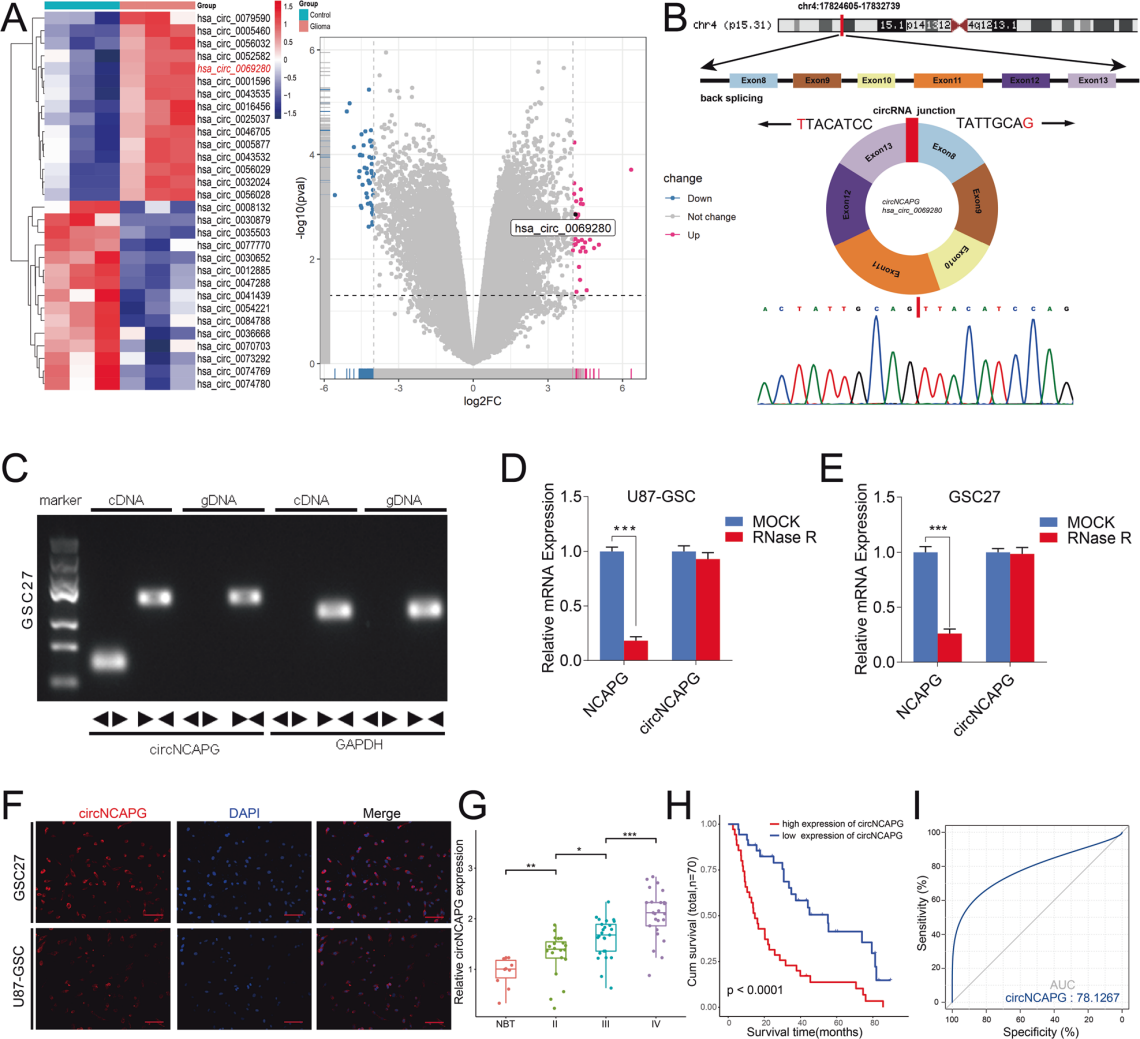
CircNCAPG is up-regulated in glioma tissues and indicates poor prognosis in glioma patients. **A** The heatmap and volcano plot showed circNCAPG was upregulated in three paired tissues of glioma patients according to GSE109569. Cut-off is |logFC | >4 and *P* < 0.05. **B** Diagram showing the formation of circNCAPG via exon8-13 back-splicing from linear NCAPG, and Sanger sequencing confirmed the backsplice junction site of circNCAPG. **C** Agarose gel electrophoresis analysis for PCR products from cDNA and gDNA. **D**, **E** The circNCAPG and NCAPG mRNA expression levels in U87-GSC and GSC27 after treatment with RNase R. **F** RNA FISH showed the localization of circNCAPG. Scale bar = 50 µm. **G** The expression of circNCAPG in NBTs and different WHO grade glioma samples. (NBT, *n* = 10; WHO grade II, *n* = 20; WHO grade III, *n* = 25; WHO grade IV, *n* = 25). **H** Kaplan–Meier analysis of 70 glioma patients determined the relationship of circNCAPG expression level and overall survival. **I** ROC curve of circNCAPG expression in 70 glioma patients. All results are presented as the mean ± SD (three independent experiments). *p < 0.05; **p < 0.01; ***p < 0.001.

To investigate the differential expressed circRNAs in glioma, we analyzed GSE109569 dataset which contained three paired GBM tissues. A total of 91 differentially expressed circRNAs were identified with fold change >4.0 and *p* < 0.05. The results showed that hsa_circ_0069280 (circNCAPG) was upregulated in GBM (Fig. 1A). CircNCAPG is generated by back splicing from exons 8–13 of the NCAPG gene according to circBase database annotation. Sanger sequencing confirmed the backsplice junction site of circNCAPG (Fig. 1B). Then we used qPCR to detect circNCAPG expression between glioma differentiated cells and glioma stem cells derived from U87, U251, and LN229 cell lines. The result showed that circNCAPG expression in glioma stem cells is higher than in glioma differentiated cells (Supplementary Fig. 1A). qPCR and agarose gel electrophoresis analyses demonstrated that divergent primers amplified circNCAPG in cDNA but not in genomic DNA (gDNA) (Fig. 1C). The circular structure of circNCAPG was confirmed in U87-GSC and GSC27 with RNase R treatment. The results demonstrated that the linear RNA NCAPG decreased significantly, but circNCAPG was resistant to RNase R treatment and revealed an inconsiderable change (Fig. 1D, E). Further RNA fluorescence in situ hybridization (FISH) was performed to determine the localization of circNCAPG, and the results indicated that circNCAPG was primarily located in the cytoplasm of U87-GSC and GSC27(Fig. 1F). In addition, we characterized the circNCAPG expression in our 70 glioma patient samples. qPCR showed that circNCAPG expression was positive correlated with WHO grade and increased in higher grade (Fig. 1G). Kaplan–Meier survival analyses showed that a higher expression of circNCAPG indicated poor outcomes in glioma patients (Fig. 1H). The receiver operating characteristic (ROC) curve showed that the associated AUC (area under the curve) value was 0.781, confirming the diagnostic value of circNCAPG (Fig. 1I).

### circNCAPG promotes the malignant phenotype of GSCs in vitro

**Fig. 2:**
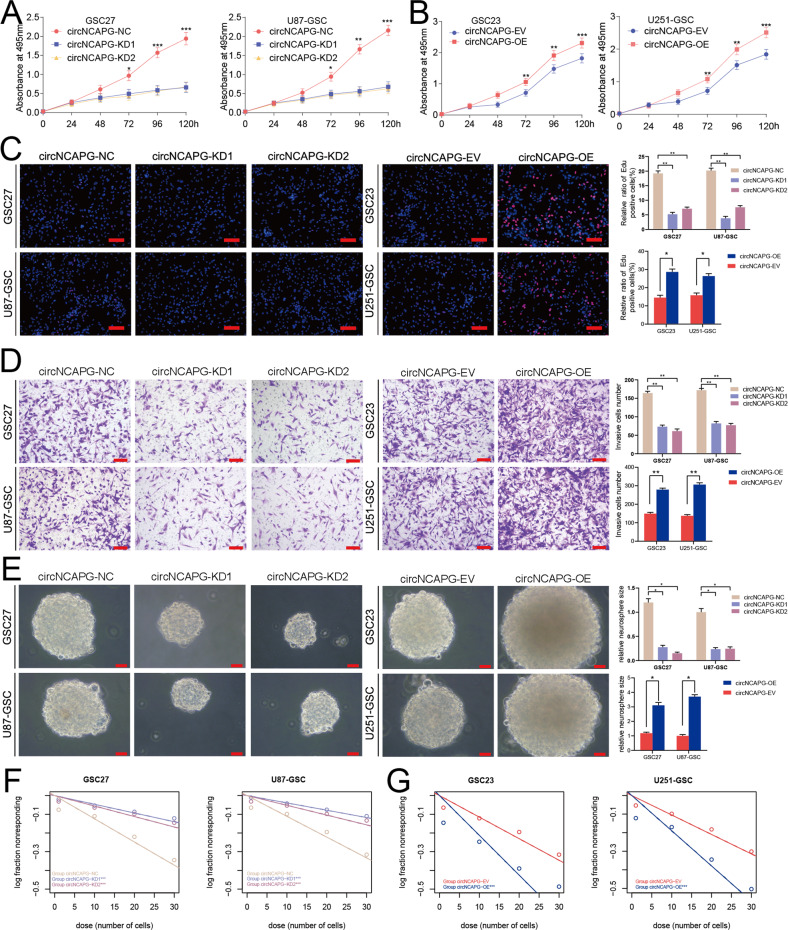
circNCAPG promotes the malignant phenotype of GSCs in vitro. **A**, **B** The MTS assays revealed the cell viability of different GSC groups after circNCAPG knockdown and overexpression treatment. **C** The Edu assays demonstrated the proliferation of different GSC groups after circNCAPG knockdown or overexpression treatment. Scale bar = 100 µm. **D** The transwell assays showed the invasive capability of GSC groups after circNCAPG knockdown or overexpression treatment. Scale bar = 50 µm. **E** The neurospheres formation assays revealed the neurospheres sizes of different GSC groups after circNCAPG knockdown or overexpression treatment. Scale bar = 20 µm. **F**, **G** The limiting dilution assays revealed the neurosphere-forming capacity of different GSC groups after circNCAPG knockdown or overexpression treatment. All results are presented as the mean ± SD (three independent experiments). *p < 0.05; **p < 0.01; ***p < 0.001.

To examine the role of circNCAPG in the malignant progression of GSCs, we selected U87-GSC and GSC27 for RNAi-mediated circNCAPG knockdown, as well as U251-GSC and GSC23 for circNCAPG overexpression. The MTS assay and EDU assay were used to measure the effect of circNCAPG on the proliferative capacity of GSCs in vitro. The results showed that circNCAPG knockdown significantly decreased U87-GSC and GSC27 cell proliferation. In contrast, the overexpression of circNCAPG promoted the proliferation of U251-GSC and GSC23 cells (Fig. 2A–C). The transwell assay showed that circNCAPG expression modification significantly altered cell invasion capability. Compared with controls, knockdown of circNACPG significantly diminished invasive ability in GSC27 and U87-GSC cells, but markedly increased invasive ability in U251-GSC and GSC23 overexpressing cells. (Fig. 2D). Further neurosphere formation assay and limiting dilution neurosphere formation assay showed that the sphere formation capacity in circNCAPG knockdown GSCs was obviously diminished, whereas it increased dramatically in circNCAPG-overexpressed GSCs (Fig. 2E–G). Together, these results suggest that circNCAPG can promote the malignant phenotype of GSCs in vitro.

### U2AF65 can bind to circNCAPG in GSCs

**Fig. 3:**
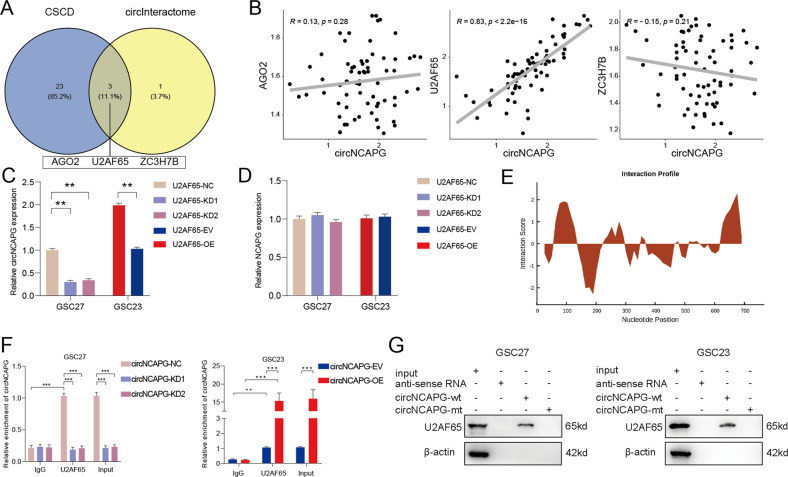
U2AF65 can bind to circNCAPG in GSCs. **A** The predictions of RNA-binding proteins for circNCAPG are based on CSCD and the circInteractome database. **B** The gene expression correlation analysis between AGO2, U2AF65, ZC3H7B and circNCAPG. **C**, **D** The qPCR assay revealed the changes of circNCAPG and NCAPG mRNA levels in different GSC groups after U2AF65 knockdown or overexpression treatment. **E** The predicted binding site between circNCAPG and U2AF65 according to the catRAPID database. **F** The RIP assays revealed enrichment levels of circNCAPG in different GSC groups. **G** The western blotting revealed that the U2AF65 protein immunoprecipitated with circNCAPG in the RNA pull-down assays. All results are presented as the mean ± SD (three independent experiments). *p < 0.05; **p < 0.01; ***p < 0.001.

Previous research has demonstrated that RBP proteins could bind to and regulate circRNA expression [25, 26]. We searched CSCD and circInteractome databases to find potential circNCAPG binding proteins. The predicted results showed that AGO2, U2AF65, and ZC3H7B may interact with circNCAPG (Fig. 3A). Then we used our patient samples to explore gene expression correlation between AGO2, U2AF65, ZC3H7B, and circNCAPG. The correlation analysis of qPCR results relieved that U2AF65 is the most probable binding protein for circNCAPG (Fig. 3B). We then established U2AF65 knockdown and overexpression GSC cell models. qRT-PCR demonstrated that circNCAPG expression was visibly suppressed in GSC27 cells with U2AF65-knockdown and significantly increased in GSC23 cells with U2AF65-overexpression, but there was no remarkable alteration in linear NCAPG expression (Fig. 3C, D). Moreover, RIP assays were performed by using previously established circNCAPG knockdown and overexpression GSC models to detect whether U2AF65 can bind to circNCAPG. qRT-PCR demonstrated that the enrichment levels of circNCAPG were dramatically increased in the anti-U2AF65 group compared to the IgG group. The enrichment levels of circNCAPG were significant decreased in GSC27 with circNCAPG-knockdown, while circNCAPG enrichment levels were increased in GSC23 with circNCAPG-overexpression (Fig. 3F). According to the catRAPID database, the U2AF65 protein is most probably bound to the 91-142 region of circNCAPG (Fig. 3E). RNA pull-down assay exhibited that U2AF65 protein was pulled down with the biotinylated wild-type circNCAPG probe in both GSC27 and GSC23, whereas the circNCAPG-mt probe could not (Fig. 3G). In summary, the preceding results indicate that U2AF65 interacts with circNCAPG.

### U2AF65 regulates the malignant progression of GSCs in vitro via maintaining the stability of circNCAPG

To confirm the mechanisms whereby U2AF65 upregulates the expression of circNCAPG. We then examined the stability of circNCAPG and the result proved that the circNCAPG stability was decreased in the control group compared to the U2AF65 overexpression group (Supplementary Fig. 1B). MTS, EDU, transwell, neurosphere formation and limiting dilution neurosphere formation assays were performed and the results indicated that U2AF65 overexpression dramatically promoted proliferation, invasion and sphere formation capabilities of GSCs. However, circNCAPG knockdown remarkably reversed these phenotypes (Supplementary Fig. 1C–G). Taken together, U2AF65 promotes the malignant progression of GSCs via maintaining the stability of circNCAPG in vitro.

### circNCAPG can interact with and enhance the nuclear translocation of RREB1

**Fig. 4:**
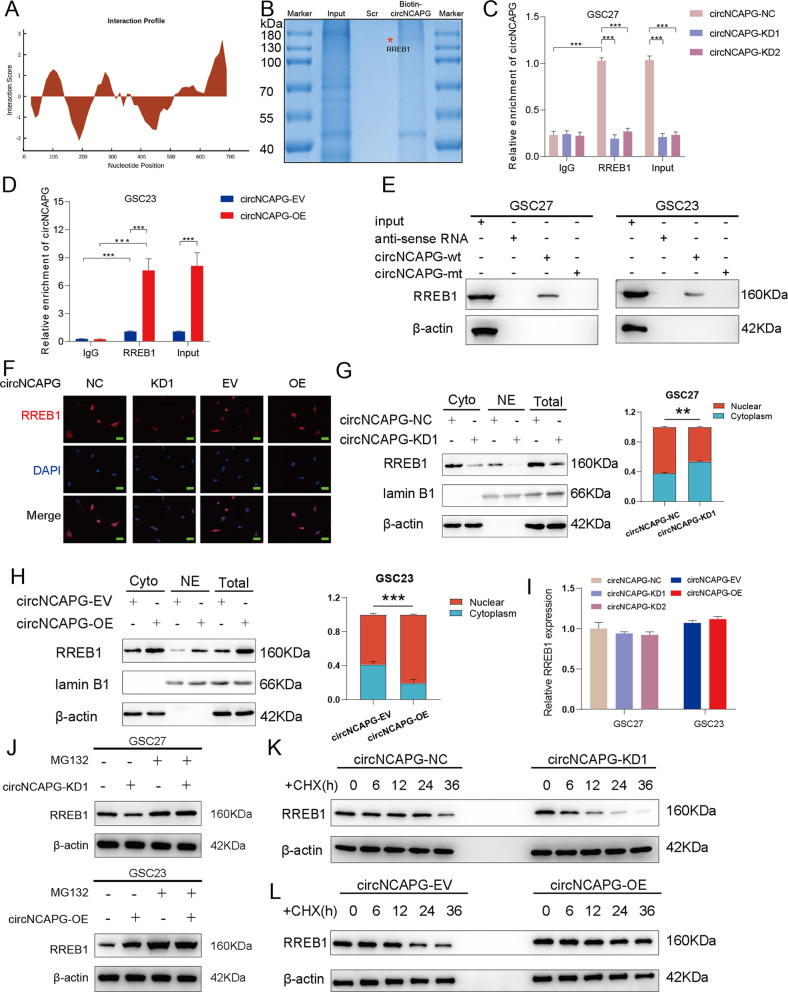
circNCAPG can interact with and enhance the nuclear translocation of RREB1. **A** The catRAPID database predicted binding sites between circNCAPG and RREB1. **B** LC-MS/MS identified the interacting proteins of circNCAPG after pull-down with biotin-labeled circNCAPG probe. **C**, **D** The RIP assays revealed circNCAPG enrichment levels in different GSC groups. **E** The western blot and RNA pull down revealed that the circNCAPG interacts with RREB1 protein. **F** Immunofluorescence detected the distribution of RREB1 protein in GSCs with circNCAPG knockdown or overexpression. Scale bars = 50 µm. **G**, **H** Western blotting detected the RREB1 protein levels and proportions in the nuclear and cytoplasmic lysates in GSCs with circNCAPG knockdown or overexpression. **I** qPCR revealed RREB1 mRNA levels in GSCs with circNCAPG-KD or circNCAPG-OE. **J** The western blotting revealed RREB1 protein levels in GSCs with circNCAPG knockdown or overexpression treated with MG132 (50 µM) for 6 h. **K**, **L** Protein levels of RREB1 were detected by western blotting in GSCs with circNCAPG knockdown or overexpression treated with cycloheximide (CHX, 100 ng/ml). All results are presented shown as the mean ± SD (three independent experiments). *p < 0.05; **p < 0.01; ***p < 0.001.

Since circRNAs can interact with and regulate intracellular localization of transcription factors to exert their functions in tumor [29]. We found that transcription factor RREB1 is the most potential transcription factor that interacts with circNCAPG according to the catRAPID database (Fig. 4A and Supplementary Table 3). The potential binding proteins of circNCAPG were detected by LC-MS/MS (Fig. 4B and Supplementary Table 4). Combined with the prediction of catRAPID database, we selected RREB1 for further study. Then the RIP assays demonstrated that circNCAPG enrichment levels in the anti-RREB1 group were significantly higher compared to the treatment with IgG group. Moreover, circNCAPG enrichment levels were considerably weakened in GSC27 with circNCAPG-knockdown compared to the control group, while the enrichment levels of circNCAPG were increased in GSC23 with circNCAPG-overexpression (Fig. 4C, D). RNA pulldown assays also indicated that circNCAPG-wt could interact with RREB1 in both GSC27 and GSC23, not circNCAPG-mt (Fig. 4E). Further, we detected the distribution of RREB1 in GSCs via the immunofluorescence and western blotting (Fig. 4F–H). The results indicated that RREB1 was considerably reduced within the nucleus of the circNCAPG knockdown, but increased in the circNCAPG overexpression. Moreover, RREB1 protein expression showed similar results. These results reveal that circNCAPG interacts with and promotes the nuclear translocation of RREB1.

### circNCAPG maintains the stability of RREB1 protein

Since circNCAPG was mainly localized in the cytoplasm, we assumed that circNCAPG only regulates RREB1 protein expression. qPCR demonstrated that RREB1 mRNA expression was unchanged in both circNCAPG silencing and circNCAPG overexpression groups compared to the control group (Fig. 4I). We then found that circNCAPG knockdown remarkably reduced the protein expression level of RREB1, which could be reversed by the treatment with the proteasome inhibitor MG-132. However, RREB1 protein expression was increased after MG-132 treatment in circNCAPG overexpression group (Fig. 4J). Additionally, the half-life of RREB1 protein was shorter in the circNCAPG knockdown group following treatment with the translation inhibitor cycloheximide (CHX), whereas the circNCAPG overexpression group exhibited the opposite result (Fig. 4K, L). Altogether, these results demonstrate that circNCAPG promotes RREB1 protein expression by maintaining its stability.

### RREB1 knockdown can abolish circNCAPG induced malignant phenotype of GSCs

**Fig. 5:**
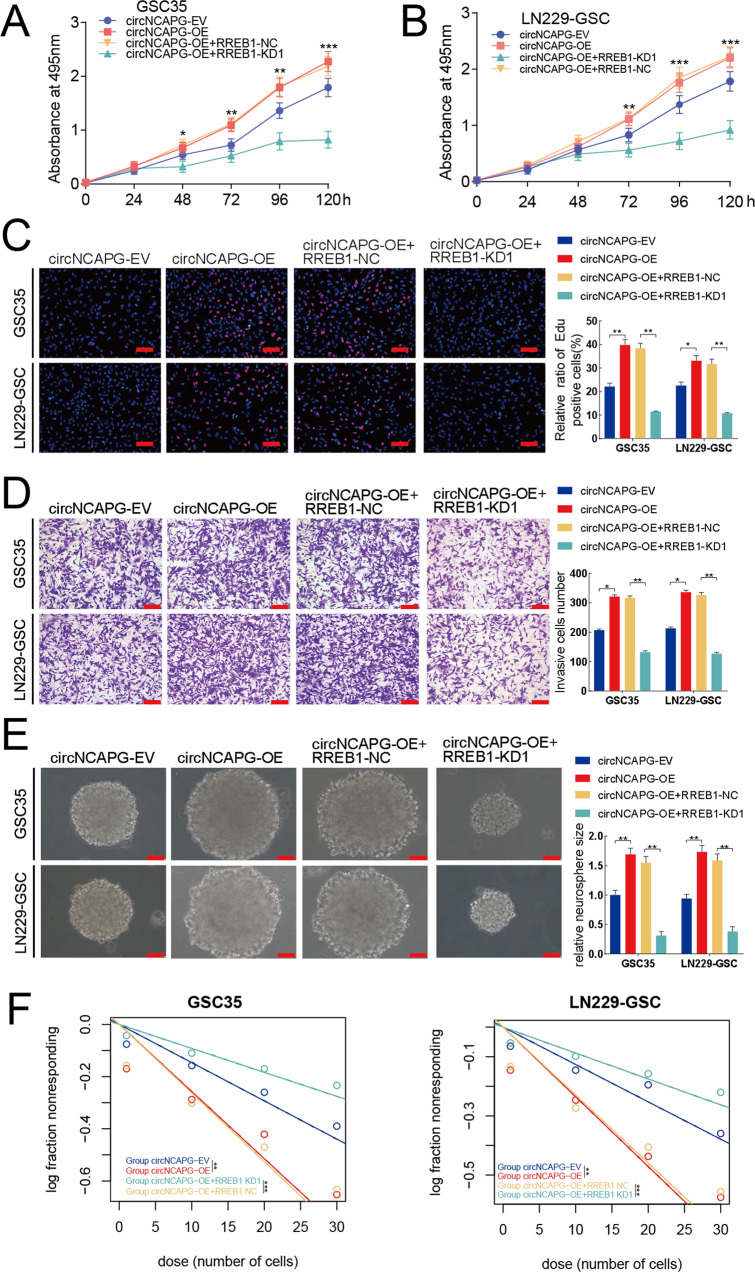
RREB1 knockdown can abolish circNCAPG induced malignant phenotype of GSCs. **A**, **B** The MTS assays revealed that circNCAPG-OE increased cell viability of GSCs and it was reversed after RREB1 knockdown treatment. **C** The EDU assays revealed the proliferation of GSCs with circNCAGPG overexpression decreased after RREB1 knockdown treatment. Scale bar = 100 µm. **D** The transwell assays revealed the invasive cells of GSCs with circNCAGPG overexpression decreased after RREB1 knockdown treatment. Scale bar = 50 µm. **E**, **F** The neurospheres formation and the limiting dilution assays showed that the sphere-forming capacity of GSCs with circNCAPG overexpression was distinctly reduced after RREB1 knockdown treatment. Scale bar = 20 µm. All results are presented as the mean ± SD (three independent experiments). *p < 0.05; **p < 0.01; ***p < 0.001.

Since circNCAPG promotes the malignant progression of GSCs and maintains the stability of RREB1 protein, we determined whether circNCAPG regulated the malignant progression of GSCs via promoting RREB1 expression. The aforementioned assays including MTT, EDU, transwell, neurosphere formation and limiting dilution neurosphere formation were then performed on GSC35 and LN229-GSC. The results showed that the proliferation, invasion, and sphere formation capabilities of circNCAPG-overexpressed GSCs were significantly diminished after RREB1 knockdown (Fig. 5A–F). Therefore, these results suggested that RREB1 knockdown can abolish malignant phenotype of GSCs induced by circNCAPG in vitro.

### RREB1 transcriptionally upregulates U2AF65 and NESTIN expression and maintains the stemness of GSCs

**Fig. 6:**
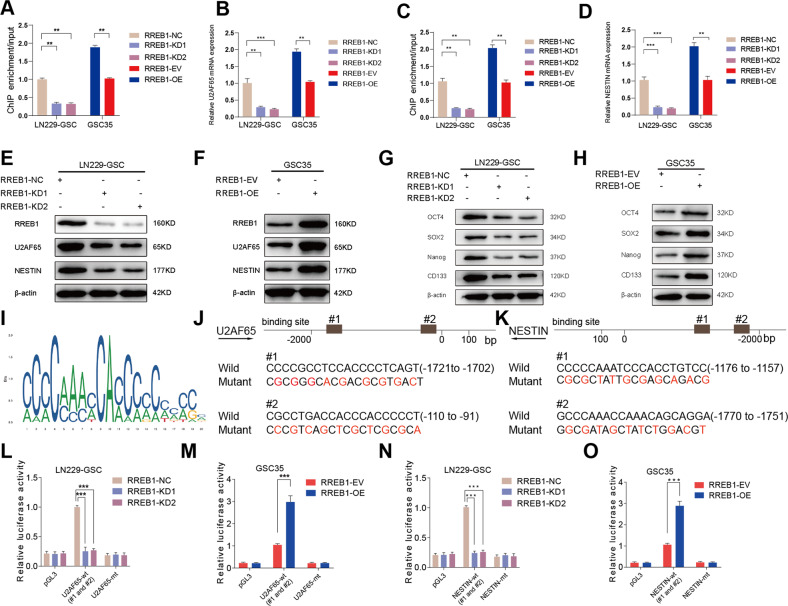
RREB1 transcriptionally upregulates U2AF65 and NESTIN expression and maintains the stemness of GSCs. **A**, **C** The ChIP qPCR assays revealed that RREB1 directly binds to the promoter of U2AF65 (**A**) and NESTIN (**C**). **B**, **D** The qPCR assays revealed the mRNA expression levels of U2AF65 (**B**) and NESTIN (**D**) in GSCs with RREB1 knockdown and overexpression treatment. **E**, **F** The western blotting revealed RREB1, U2AF65, NESTIN protein levels in GSCs with RREB1 knockdown (**E**) and overexpression (**F**) treatment. **G**, **H** Stemness markers were detected via western blotting in GSCs with RREB1 knockdown (**G**) and overexpression (**H**) treatment. **I** The diagram of RREB1 binding motif (JASPAR database). **J**, **K** The presumed RREB1 binding site in U2AF65 (**J**) and NESTIN (**K**) promoter. **L**–**O** The luciferase reporter assays revealed that RREB1-KD or RREB1-OE influenced the luciferase promoter activities of U2AF65 (**L**, **M**) and NESTIN (**N**, **O**). All results are presented as the mean ± SD (three independent experiments). *p < 0.05; **p < 0.01; ***p < 0.001.

Since RREB1 is a transcription factor, we subsequently surveyed that whether RREB1 transcriptionally regulates U2AF65 and stemness marker genes expression in GSCs. We explored the gene expression correlation between RREB1 and U2AF65, NESTIN, CD133, Nanog, OCT4, and SOX2 in TCGA dataset from GEPIA2 database. The spearman correlation analyses showed that U2AF65 was highly positively correlated with U2AF65 (Supplementary Fig. 2A). The stemness marker gene NESTIN was most positively correlated with RREB1 (Supplementary Fig. 2B, C). Then we performed ChIP to confirm whether RREB1 can directly transcriptionally upregulate the expression of U2AF65 and stemness marker genes. The ChIP assays revealed that, in contrast to the control group, the enrichment of U2AF65 and NESTIN was decreased in the RREB1 silencing groups whereas it was increased in the RREB1 overexpression groups (Fig. 6A, C). However, neither the RREB1 silencing group nor the RREB1 overexpression group observed a change in the enrichment of CD133, Nanog, OCT4, or SOX2 (Supplementary Fig. 2D). Additionally, qPCR assays demonstrated that RREB1 knockdown decreased the expression of U2AF65, NESTIN, but RREB1 overexpression increased it (Fig. 6B, D). However, western blotting assays showed that RREB1 knockdown downregulated the protein levels of U2AF65 and these five stemness marker genes, while RREB1 overexpression upregulated them (Fig. 6E–H). These results indicated that RREB1 can regulate the transcription of U2AF65 and NESTIN directly, but CD133, Nanog, OCT4 and SOX2 may be regulated by RREB1 via other mechanisms. Then, based on the predictions from the Jaspar database, we constructed U2AF65 and NESTIN mutant luciferase reporter plasmids, each of which contains two mutant sites (#1 and #2) (Fig. 6I–K). The luciferase reporter results showed that the luciferase activities of U2AF65-wt and NESTIN-wt were reduced in LN229-GSC with RREB1 silencing, but they were increased in GSC35 with RREB1 overexpression. Neither pGL3-mt changed significantly (Fig. 6L–O). Altogether, our results suggest that RREB1 could transcriptionally upregulate U2AF65 and NESTIN expression and maintain the stemness of GSCs in vitro.

### TGF-β1 signaling pathway is functional downstream intermediary for RREB1 in promoting the malignant phenotype of GSCs

**Fig. 7:**
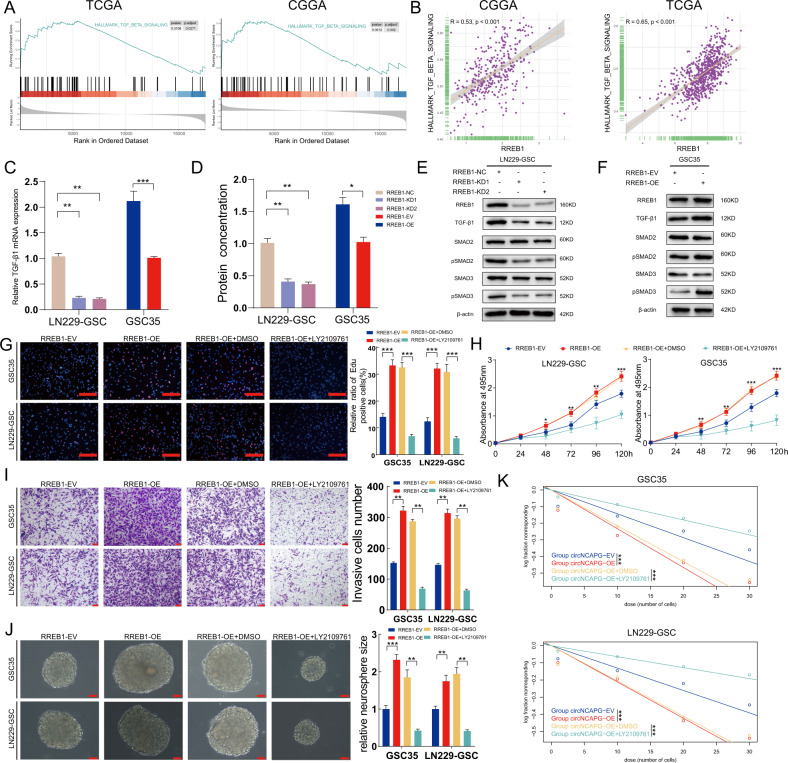
TGF-β1 signaling pathway is functional downstream intermediary for RREB1 in promoting the malignant phenotype of GSCs. **A** GSEA analysis revealed that the HALLMARK_TGF_BETA_SIGNALING signature was obvious enriched in higher RREB1 expression group in TCGA and CGGA cohorts. **B** The correlation between the HALLMARK_TGF_BETA_SIGNALING signature enrichment score assessed via ssGSEA and RREB1 expression in TCGA and CGGA cohorts. **C** The qPCR assays revealed the mRNA expression of TGF-β1 in GSCs with RREB1 knockdown and overexpression treatment. **D** The ELISA showed the protein concentration of TGF-β1 in GSCs with RREB1 knockdown and overexpression treatment. **E**, **F** The western blotting showed the protein expression levels of TGF-β1 pathway after RREB1 knockdown (**E**) and overexpression (**F**) treatment. **G**–**K** The MTS (**H**), EDU (**G**), transwell (**I**), neurosphere formation (**J**), limiting dilution neurosphere formation (**K**) showed that RREB1 affected the malignant phenotype of GSCs and was reversed by LY2109761 treatment. All results are presented as the mean ± SD (three independent experiments). *p < 0.05; **p < 0.01; ***p < 0.001.

It has been shown that RREB1 is a crucial partner of TGF-β pathways in promoting cancer progression [30]. We then used gene set enrichment analysis (GSEA) and single sample gene set enrichment analysis(ssGSEA) to investigate the correlation between RREB1 and TGF-β related signatures from the Msigdb, Biocarta Pathways, KEGG and Wikipathways databases in TCGA and CGGA cohorts. The results showed a strong positive correlation between RREB1 and HALLMARK_TGF_BETA_SIGNALING signature (Fig. 7A, B). Besides, The GSEA analysis results showed that RREB1 high group was enriched in WP_TGFBETA_SIGNALING_PATHWAY in TCGA cohort. While in CGGA cohort, RREB1 high group was enriched in the signature from Wikipathways (Supplementary Fig. 3A, C). Furthermore, the spearman correlation analysis showed that the scores of all these signatures were positively correlated to RREB1 expression (Supplementary Fig. 3B, D). Consistently, qPCR, ELISA and western blotting assays also exhibited that the TGF-β1 expression levels were diminished in GSCs with RREB1 knockdown, whereas RREB1 overexpression increased the expression of TGF-β1 in GSCs (Fig. 7C–F). Besides, western blotting showed that the expression levels of TGF-β1 signaling pathway proteins pSMAD2 and pSMAD3 were significantly down-regulated in GSCs with RREB1 knockdown, while the opposite results were observed in GSCs with RREB1 overexpression (Fig. 7E, F).

We also treated GSCs with LY2109761, a TGF-β inhibitor, to verify that RREB1 regulated the malignant phenotype of GSCs via activating TGF-β1 signaling pathway. The results showed that LY2109761 treatment remarkably reversed the enhanced proliferation and invasion abilities caused by RREB1 overexpression (Fig. 7G–I). Moreover, similar results were observed in the neurosphere formation and limiting dilution neurosphere formation assays with LY2109761 treatment (Fig. 7J, K).

TGF-β signaling pathway has been reported to regulate GSC stemness [31]. Combined with the previous results, we further explored NESTIN and TGF-β signaling pathway which one is more important for sustaining GSCs stemness. GSC35 cells were treated with NESTIN knockdown or TGF-β inhibitor LY2109761. Neurosphere formation and LDA assays showed that the neurosphere formation capacity decreased in RREB1-OE + NESTIN-KD group compared to RREB1-OE + LY2109761 group (Supplementary Fig. 4A–C). Therefore, these results indicate that RREB1 activates TGF-β1 signaling pathway to promote the malignant phenotypes and NESTIN is more important than RREB1-activated TGF-β signaling pathway for the maintenance of stemness in GSCs.

### The U2AF65/circNCAPG/RREB1 feedback loop can regulate glioma tumorigenesis in vivo

**Fig. 8:**
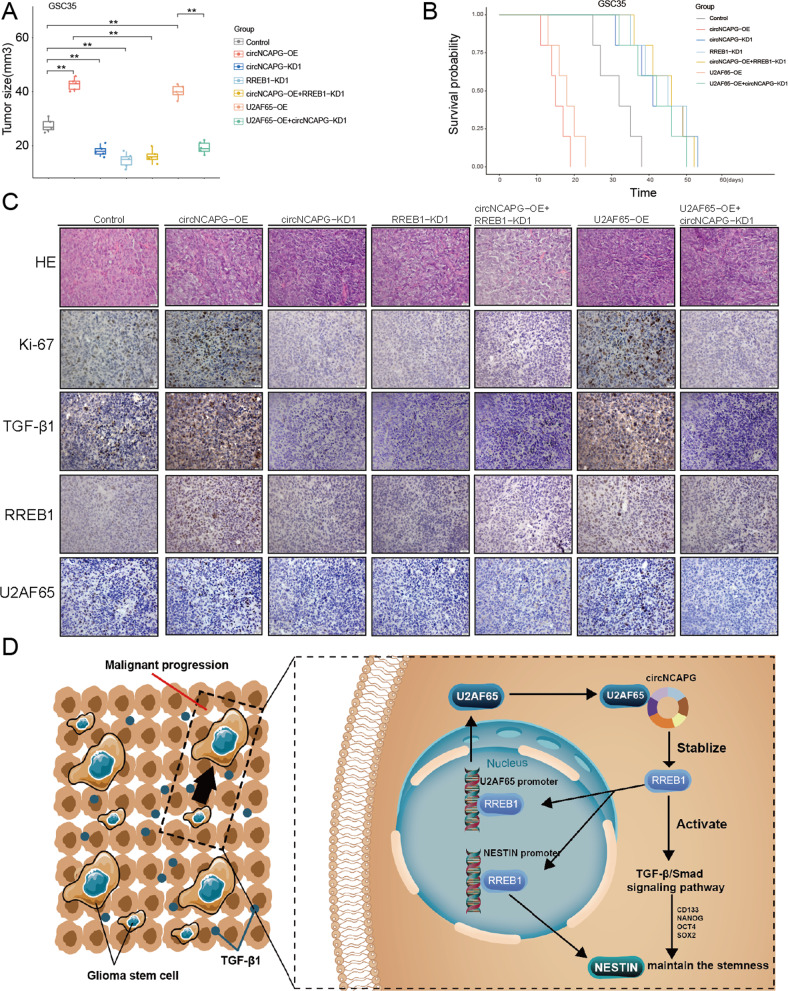
The U2AF65/circNCAPG/RREB1 feedback loop can regulate glioma tumorigenesis in vivo. **A** The tumor size of nude mice with control, circNCAP-OE, circNCAPG-KD1, RREB1-KD1, circNCAPG-OE combined with RREB-KD1, U2AF65-OE and U2AF65-OE combined with circNCAPG-KD1 groups. **B** The Kaplan–Meier survival curves in different mice groups. **C** Representative immunohistochemical staining showing the changes in Ki67, TGF-β1, RREB1 and U2AF65, and HE staining in different mice groups. **D** The schematic of the mechanisms of the U2AF65/circNCAPG/RREB1 feedback loop promotes glioma tumorigenesis via activating the TFG-β1 signaling pathway. All results are presented as the mean ± SD (three independent experiments). *p < 0.05; **p < 0.01; ***p < 0.001.

Finally, we detected the effect of the U2AF65/circNCAPG/RREB1 axis on glioma progression by establishing orthotopic xenograft models. Tumors were larger in the circNCAPG overexpression and U2AF65 overexpression groups compared to the control groups, whereas they were smaller in the circNCAPG knockdown and RREB1 knockdown groups (Fig. 8A). In addition, the combination of circNCAPG overexpression and RREB1 knockdown significantly decreased circNCAPG overexpression tumor volumes (Fig. 8A). The increased tumor volume in the U2AF65 group was similarly reduced by circNCAPG knockdown (Fig. 8A). The Kaplan–Meier survival analysis revealed a similar result. The median survival times of the circNCAPG-OE and U2AF65-OE groups were shorter compared with the control group, while they were longer in the circNCAPG knockdown, RREB1 knockdown, circNCAPG overexpression combed with RREB1 knockdown, and the combination of U2AF65 overexpression and circNCAPG knockdown groups (Fig. 8B). As demonstrated by immunohistochemistry (IHC), the expression of U2AF65, RREB1 and TGF-β1 was upregulated in the circNCAPG overexpression and U2AF65 overexpression groups compared to the control groups, while it was decreased in the circNCAPG knockdown, RREB1 knockdown, circNCAPG overexpression combed with RREB1 knockdown, and the combination of U2AF65 overexpression and circNCAPG knockdown groups (Fig. 8C). Meanwhile, the expression of ki67, which represents the degree of tumor malignancy, and TFG-β1 showed the similar results (Fig. 8C). As illustrated in Fig. 8D, the U2AF65/circNCAPG/RREB1 feedback loop stimulates glioma tumorigenesis by activating the TFG-β1 signaling pathway. Overall, the above results suggest that the U2AF65/circNCAPG/RREB1 axis can regulate glioma tumorigenesis in vivo.

## Discussion

CircRNAs are newly discovered non-coding RNAs with highly stable that are abundant in the brain [32]. Researchers have recently demonstrated that circRNAs participate in malignancy progression and are associated with poor prognosis in glioma patients [13, 14, 27, 33]. In this study, we firstly confirmed that hsa_circ_0069280, which was derived from the NCAPG gene, was remarkably upregulated in glioma patients, especially in high grade gliomas. Furthermore, Kaplan–Meier survival analyses and ROC-curve suggest that circNCAPG could potential serve as a new biomarker for glioma diagnosis and prognosis. Glioma stem cells (GSCs) are low differentiated and drive the aggressive progression of gliomas. GSCs are considered to play an important role in supporting glioma proliferation, invasion, angiogenesis, drug resistance, and recurrence [34, 35]. Increasing evidences suggested that circRNAs are crucial for maintaining the stemness and malignant phenotype of GSCs [16, 27, 36]. Therefore, we explored the effect of circNCAPG in GSCs. Elevated circNCAPG expression promoted proliferation, invasion, and maintained self-renewal in GSCs, while silencing circNCAPG suppressed these malignant phenotypes. CircNCAPG may be a novel prognostic biomarker and a molecular therapeutic target for glioma patient.

RNA-binding protein (RBP) has been demonstrated to regulate gene expression in posttranscriptional regulation, including pre-mRNA alternative splicing, mRNA translation and protein stability [37]. Multiple studies have demonstrated that RBP also regulates circRNA expression [38]. For example, the RNA-binding protein FUS could binds to flanking introns around the circularized exons to regulate the production of 19 circRNAs [39]. In our study, bioinformatics analysis predicted AGO2, U2AF65, and ZC3H7B as candidate RBPs for circNCAPG, and we confirmed that U2AF65 binds to circNCAPG in GSCs and promotes its stability and expression.

It is reported that U2AF65 functionally promote drug resistance in several tumors [40, 41]. Our previous study identified that RNA-binding protein U2AF2 can promote the progression of GSCs via stabilizing cARF. However, it is uncertain whether U2AF65 influences GSCs progression. We also confirmed that U2AF65 can enhance GSCs malignancy in this study, but that this impact can be reversed by circNCAPG knockdown, as demonstrated in functional assays. circNCAPG is downstream functional molecule for U2AF65.

Currently, studies on circRNAs has mainly focused on the competing endogenous RNA (ceRNA) mechanism. Hsa_circ_0007244, an another circRNA generated from NCAPG, act as miRNA sponge on glioma proliferation and invasion [42]. However, increasing studies suggested that circRNAs can also perform biological functions by binding directly to proteins, regulating protein- protein interaction, altering the cellular localization of transcription factors, and even can be translated [10, 29, 43]. We focused on the relationship between circNCAPG with transcription factors in glioma. RREB1 was the most probable candidate transcription factor that interacts with circNCAPG according to the prediction of catRAPID database. Further assays confirmed that circNCAPG can directly bind to and promote the nuclear translocation of RREB1. circNCAPG promoted RREB1 protein expression level by stabilizing RREB1, but not by gene transcription. In addition, RREB1 knockdown blocked the function of circNCAPG in promoting GSCs progression. Surprisingly, we found RREB1 transcriptionally regulates U2AF65 and NESTIN expression, which respectively regulate circNCAPG expression and maintains GSCs stemness. Therefore, a feedback loop U2AF65/circNCAPG/RREB1 was constructed in GSCs. Unexpectedly, we found that RREB1 did not directly regulate the transcription of CD133, Nanog, OCT4, and SOX2 genes. However, RREB1 upregulated the protein levels of CD133, SOX2, Nanog, and OCT4. Moreover, the RBP prediction showed that AGO2 is one of the candidate RNA-bind protein. Although AGO2 and circNCAPG were not highly correlated in correlation analyses, AGO2 is an essential protein involved in miRNA formation and mediates ceRNA mechanism of circRNAs [44]. The mechanism by which circNCAPG promotes glioma progression requires further investigation.

It is reported that RREB1 has been implicated in the regulation of tumorigenesis and progression in a number of cancers [18, 45, 46]. However, the mechanism of RREB1 in promoting tumor progression in glioma is unclear. RREB1 has been observed to act as an essential partner in the induction of EMT by the TGF-β1 pathway in tumors [30]. Increasing evidences show that TGF-β1 pathway was activated to promote the biological progression of glioma [47, 48]. We further explored the correlation between RREB1 and TGF-β1 signaling pathway related signatures, and confirmed that the HALLMARK_TGF_BETA_SIGNALING signature enriched in higher RREB1 group. The TGF-β inhibitor LY2109761 could abolish RREB1 induced malignant phenotype of GSCs. We confirmed that mechanistically RREB1 enhances the malignant progression of GSCs by activating the TGF-β pathway. Further, RREB1 can directly regulate NESTIN gene expression which plays an important role as a glioma stem cell biomarker in maintaining glioma stemness [49]. However, stemness markers CD133, Nanog, OCT4, SOX2 protein levels also upregulated by RREB1. It is reported that TGF-β pathway contributes to GSC stemness [50]. The activation of αvβ8 integrin-TGFβ1 signaling axis maintains GSC stemness via upregulation of CD133 and SOX2 expression [51]. Prrx1 enhances the acquisition of stemness in non-stem tumor cells and maintains GSC stemness by promoting CD133 and SOX2 expression through activation of TGFβ pathway [31]. Compared to the activation of TGF-β pathway by RREB1, we observed that NESTIN was more effective at sustaining neurosphere formation capacity, indicating that NESTIN plays a more important role in maintaining GSC stemness.

Finally, in vivo experiments revealed that the U2AF65/circNCAPG/RREB1 feedback loop also regulates glioma progression in the orthotopic intracranial glioma model. Ki67, a cell proliferation marker, and TGF-β1 expression also followed the altered U2AF65/circNCAPG/RREB1 feedback loop. The potential clinical value of the U2AF65/circNCAPG/RREB1 axis in GBM is highlighted by these observations.

In conclusion, our study clarified both the upstream regulation and downstream function of circNCAPG. U2AF65 can directly bind and stabilize circNCAPG, circNCAPG participates in the nuclear translocation of RREB1, a transcription factor of unknown function in GSCs, for activating the TGF-β pathway and promoting glioma progression. We broaden the understanding of circRNAs and provide a promising therapeutic target in glioma.

## Supplementary information

Supplementary legends—clean

Supplementary Figure 1

Supplementary Figure 2

Supplementary Figure 3

Supplementary Figure 4

Supplementary Table 1

Supplementary Table 2

Supplementary Table 3

Supplementary Table 4

Original Data File

aj-checklist

## Data Availability

All the data obtained for the study could be available by inquiring the corresponding author.
