# Supplementary material for: The U2AF65/circNCAPG/RREB1 feedback loop promotes malignant phenotypes of glioma stem cells through activating the TGF-β pathway
Source: Cell Death Dis. 2023 Jan 13;14(1):23. doi: 10.1038/s41419-023-05556-y (PMC9837049; doi:10.1038/s41419-023-05556-y)
Supplement: Supplementary file 1 — Supplementary legends—clean [file 41419_2023_5556_MOESM1_ESM.docx]

**Supplementary figure legends**

**Supplementary Figure 1. U2AF65 regulates the malignant progression of GSCs in vitro via maintaining the stability of circNCAPG**

A: The expression of circNCAPG in glioma stem cells and glioma differentiated cells.

B: The expression of circNCAPG under the treatment of Actinomycin D at 0,2,4,6,8,10,12h.

C: The MTS assays revealed that the cell viability of GSCs with U2AF65 overexpression can be reversed after circNCAPG knockdown treatment.

D: The limiting dilution assays revealed the neurosphere-forming capacity of GSCs with U2AF65 overexpression was diminished after circNCAPG knockdown treatment.

E: The EDU assays revealed that the proliferation the of GSCs with U2AF65 overexpression could be reversed after circNCAPG knockdown treatment.

F: The transwell invasion assays revealed that the circNCAPG knockdown treatment inhibited the invasion of GSCs with U2AF65 overexpression.

G: The neurospheres formation assays showed that the sphere-forming size of GSCs with U2AF65 overexpression was dramatically decreased after circNCAPG knockdown treatment.

All results are presented as the mean ± SD (three independent experiments). *p < 0.05; **p < 0.01; ***p < 0.001

**Supplementary Figure 2. The expression of U2AF65 and NESTIN was highly positive correlated to RREB1 expression in the TCGA dataset**

A: The correlation between U2AF65 and RREB1 expression in TCGA dataset from GEPIA2 database.

B: The correlation between NESTIN, CD133 and RREB1 expression in TCGA dataset from GEPIA2 database.

C: The correlation between Nanog, OCT4, SOX2 and RREB1 expression in TCGA dataset from GEPIA2 database.

D: The ChIP qPCR assays detected CD133, Nanog, OCT4, SOX2 promoters.

All results are presented as the mean ± SD (three independent experiments). *p < 0.05; **p < 0.01; ***p < 0.001

**Supplementary Figure 3. The correlation between RREB1 and TGF-β signaling pathway related signatures in TCGA and CGGA cohorts.**

A: GSEA analysis revealed that the WP_TGFBETA_SIGNALING_PATHWAY signature was enriched in higher RREB1 expression group in TCGA cohorts (p <0.05).

B: The correlation analyses revealed that TGF-β related signatures from the Biocarta Pathways, KEGG and Wikipathways databases were positively correlated to RREB1 in TCGA cohort (p <0.05).

C: GSEA analysis revealed that the WP_TGFBETA_SIGNALING_PATHWAY signature was enriched in higher RREB1 expression group in CGGA cohorts (p.adj<0.05).

D: The correlation analyses revealed that TGF-β related signatures from the Biocarta Pathways, KEGG and Wikipathways databases were positively correlated to RREB1. (p<0.05).

**Supplementary Figure 4. NESTIN plays more important role in sustaining stemness of GSCs.**

A, B: The neurosphere formation assays showed that the sphere-forming size of GSCs was increased in RREB1-OE+NESTIN-KD compared to RREB1-OE+LY2109761 groups.

C: The limiting dilution assays revealed the neurosphere-forming capacity of GSCs was increased in RREB1-OE+NESTIN-KD compared to RREB1-OE+LY2109761 groups.

All results are presented as the mean ± SD (three independent experiments). *p < 0.05; **p < 0.01; ***p < 0.001
