# Supplementary figures and images for: The U2AF65/circNCAPG/RREB1 feedback loop promotes malignant phenotypes of glioma stem cells through activating the TGF-β pathway
Source: Cell Death Dis. 2023 Jan 13;14(1):23. doi: 10.1038/s41419-023-05556-y (PMC9837049; doi:10.1038/s41419-023-05556-y)

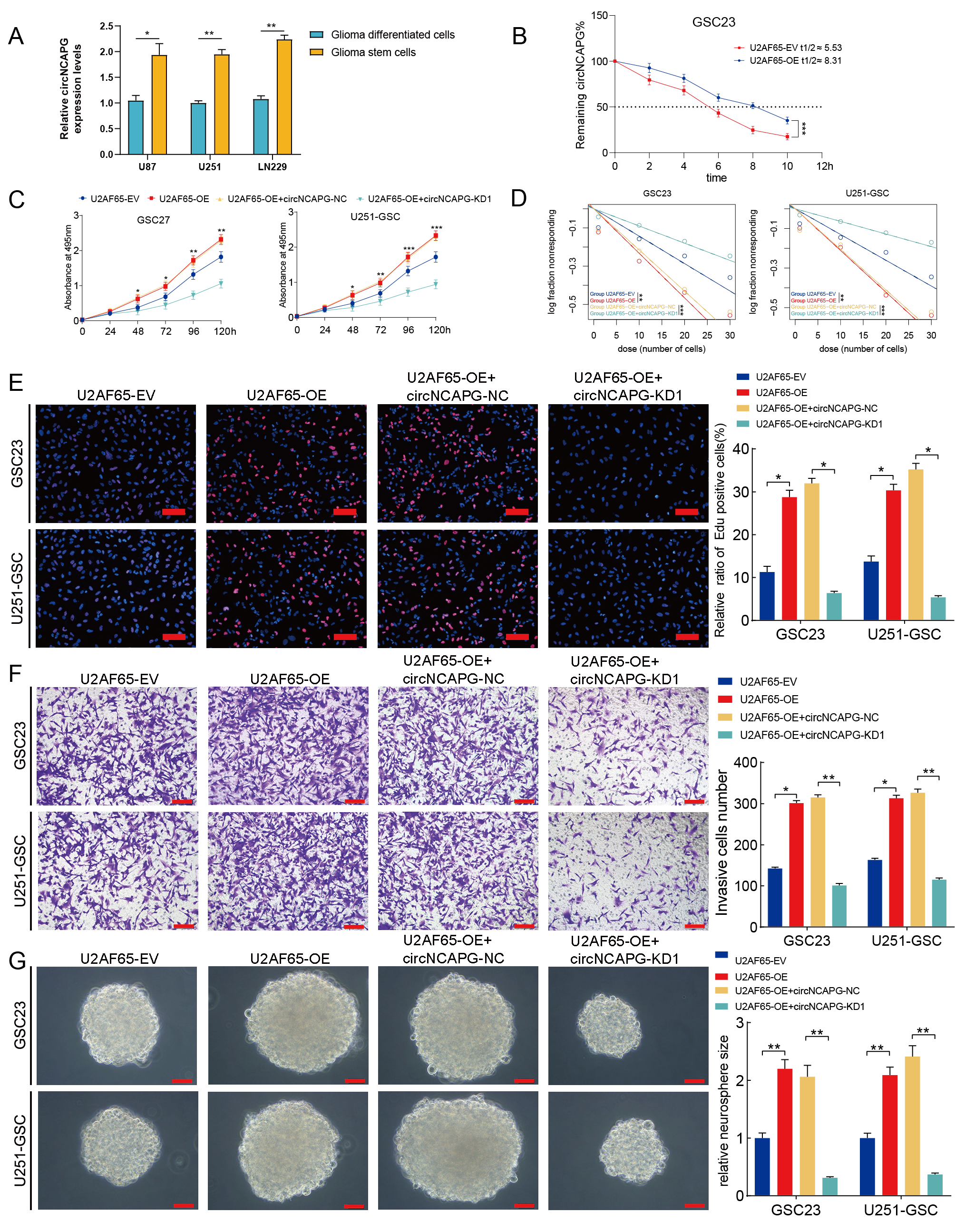

Supplement: Supplementary file 2 — Supplementary Figure 1 [file 41419_2023_5556_MOESM2_ESM.png]

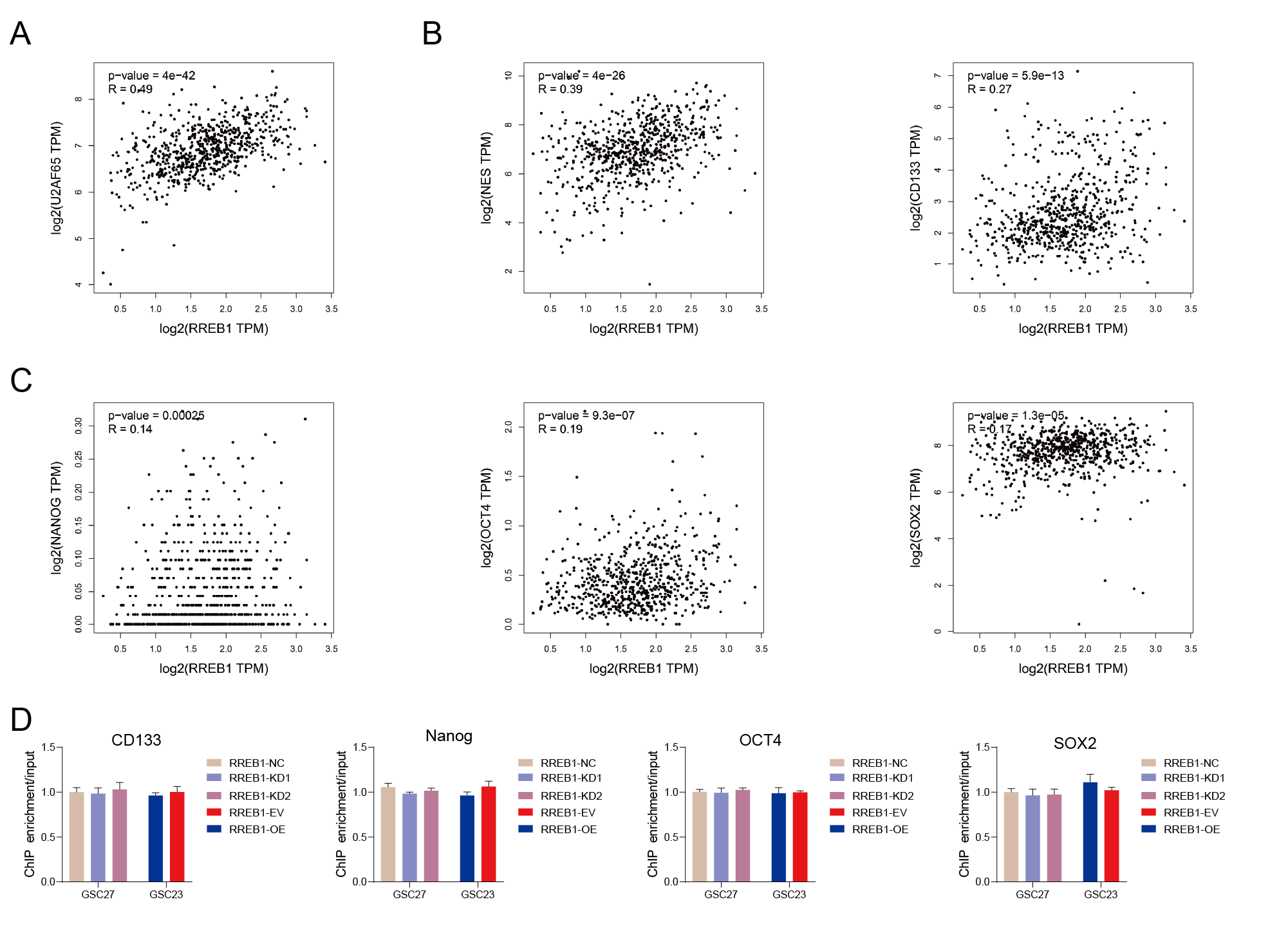

Supplement: Supplementary file 3 — Supplementary Figure 2 [file 41419_2023_5556_MOESM3_ESM.png]

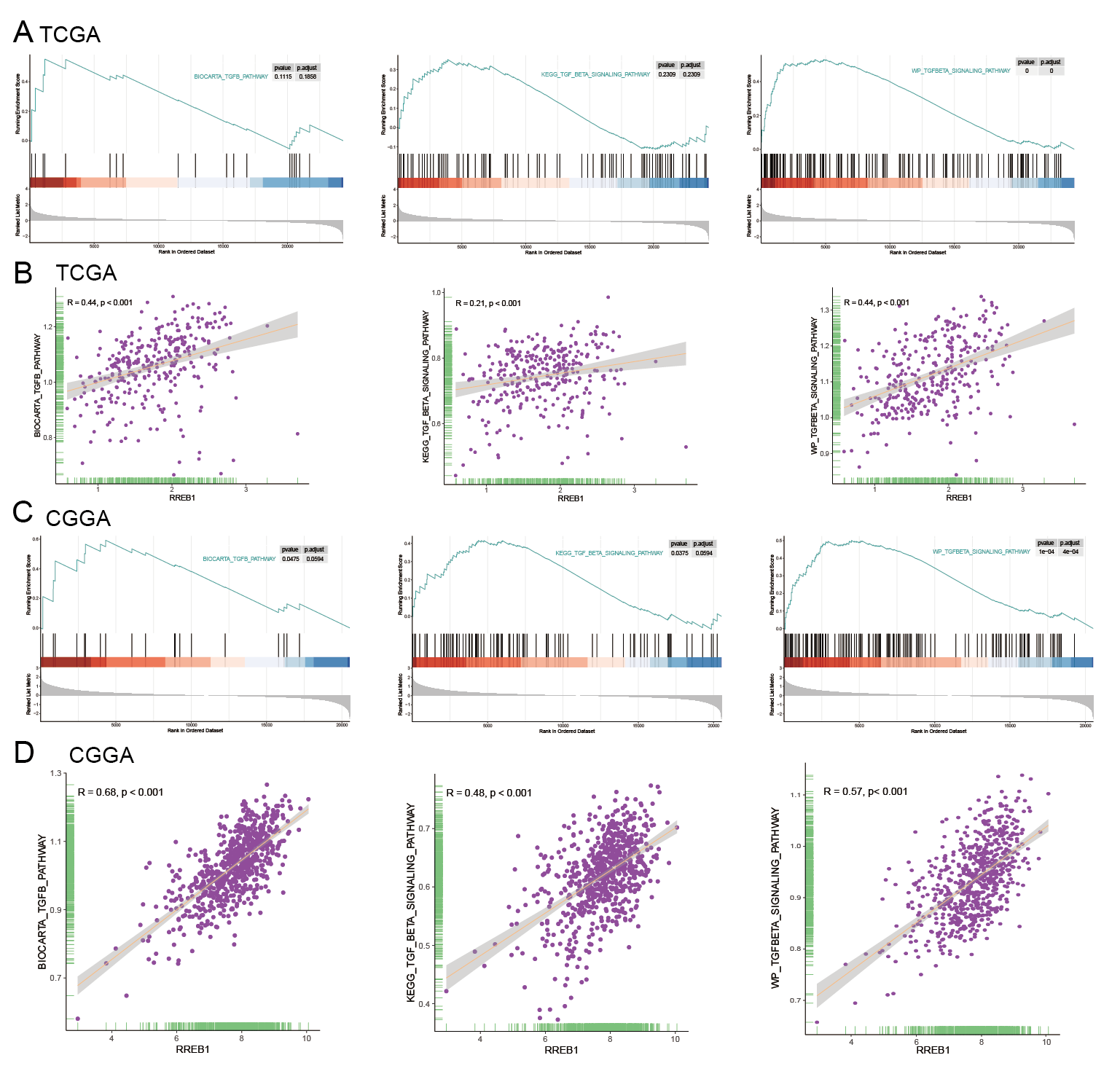

Supplement: Supplementary file 4 — Supplementary Figure 3 [file 41419_2023_5556_MOESM4_ESM.png]

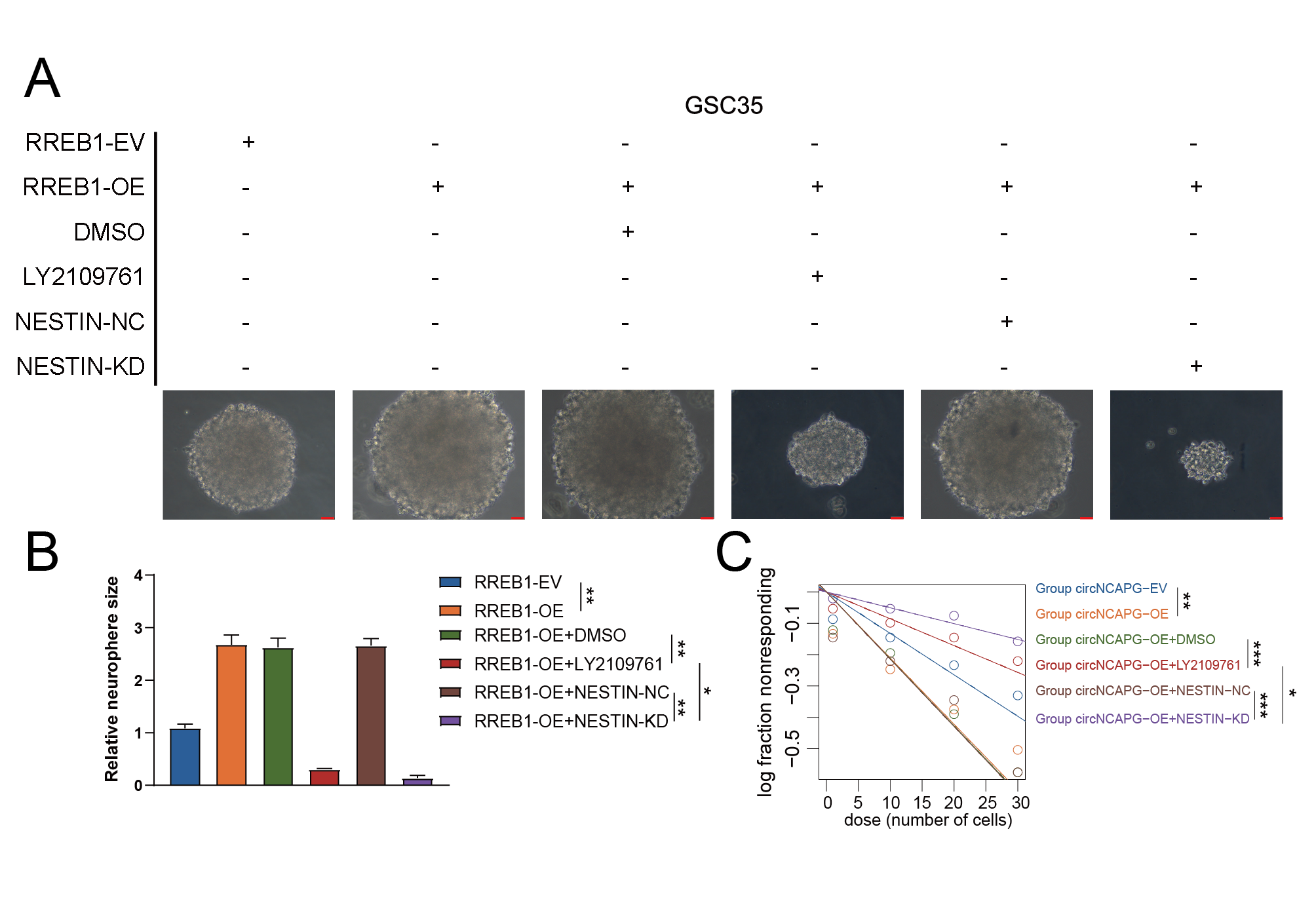

Supplement: Supplementary file 5 — Supplementary Figure 4 [file 41419_2023_5556_MOESM5_ESM.png]

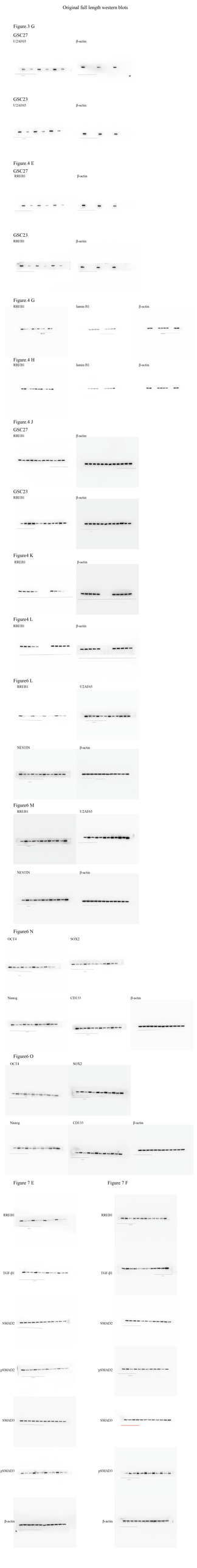

Supplement: Supplementary file 10 — Original Data File [file 41419_2023_5556_MOESM10_ESM.png]
