# Supplementary material for: The U2AF65/circNCAPG/RREB1 feedback loop promotes malignant phenotypes of glioma stem cells through activating the TGF-β pathway
Source: Cell Death Dis. 2023 Jan 13;14(1):23. doi: 10.1038/s41419-023-05556-y (PMC9837049; doi:10.1038/s41419-023-05556-y)
Supplement: Supplementary file 6 — Supplementary Table 1 [file 41419_2023_5556_MOESM6_ESM.docx]

**Table S1. The database website**

| GEO | https://www.ncbi.nlm.nih.gov/geo/ |
| --- | --- |
| TCGA | https://portal.gdc.cancer.gov/ |
| CGGA | http://www.cgga.org.cn/ |
| GSEA | https://www.gsea-msigdb.org/gsea/msigdb/index.jsp |
| CSCD | http://gb.whu.edu.cn/CSCD/ |
| circInteractome | https://circinteractome.nia.nih.gov/ |
| circBase | http://www.circbase.org/ |
| catRAPID | http://s.tartaglialab.com/page/catrapid_group |
| Jaspar | https://jaspar.genereg.net/ |
