# Supplementary material for: The U2AF65/circNCAPG/RREB1 feedback loop promotes malignant phenotypes of glioma stem cells through activating the TGF-β pathway
Source: Cell Death Dis. 2023 Jan 13;14(1):23. doi: 10.1038/s41419-023-05556-y (PMC9837049; doi:10.1038/s41419-023-05556-y)
Supplement: Supplementary file 7 — Supplementary Table 2 [file 41419_2023_5556_MOESM7_ESM.docx]

**Table S2. PCR Primers and siRNA sequences**

**qPCR Primers**

| Primer | Forward (5’-3’) | Reverse (5’-3’) |
| --- | --- | --- |
| circNCAPG | TGCTTGGGATGCTGTGGACT | CTTCATTAACAACTGGAATGCTCTGGA |
| NCAPG | GAGGCTGCTGTCGATTAAGGA | AACTGTCTTATCATCCATCGTGC |
| U2AF65 | CGGCAGCTCAACGAGAATAAA | GGGAACGAATCAGTCCACCG |
| RREB1 | AGGTTCAGACCTATCTTCCATCA | CTGCCAATCCGATTTGGTCCT |
| TGFB1 | GGCCAGATCCTGTCCAAGC | GTGGGTTTCCACCATTAGCAC |
| NESTIN | CTGCTACCCTTGAGACACCTG | GGGCTCTGATCTCTGCATCTAC |
| CD133 | AGTCGGAAACTGGCAGATAGC | GGTAGTGTTGTACTGGGCCAAT |
| NANOG | TTTGTGGGCCTGAAGAAAACT | AGGGCTGTCCTGAATAAGCAG |
| OCT4 | CTGGGTTGATCCTCGGACCT | CCATCGGAGTTGCTCTCCA |
| SOX2 | GCCGAGTGGAAACTTTTGTCG | GGCAGCGTGTACTTATCCTTCT |
| GAPDH | GGAGCGAGATCCCTCCAAAAT | GGCTGTTGTCATACTTCTCATGG |
| β-actin | CATGTACGTTGCTATCCAGGC | CTCCTTAATGTCACGCACGAT |

**siRNA sequences**

| **Primer** | **Forward (5’-3’)** | **Reverse (5’-3’)** |
| --- | --- | --- |
| circNCAPG-KD1 | UAGUAAUACGAAGUGUUUCCU | GAAACACUUCGUAUUACUAUU |
| circNCAPG-KD2 | UGUUCUUCAUUAACAACUGGA | CAGUUGUUAAUGAAGAACACA |
| U2AF65-KD1 | UUGAAGAAAUCCAUCAUGGCC | CCAUGAUGGAUUUCUUCAACG |
| U2AF65-KD2 | AUUCUUGUCCUGGUUAAUCUG | GAUUAACCAGGACAAGAAUUU |
| RREB1-KD1 | UCUUCUACCAUCUUUUUAGCU | CUAAAAAGAUGGUAGAAGACG |
| RREB1-KD2 | UCAUGAAGGCCUUGAGUUCCC | GAACUCAAGGCCUUCAUGACA |
| NESTIN-KD | ACUUUCUGGAGCUUUCUGGGA | CCAGAAAGCUCCAGAAAGUCC |
| siRNA-NC | UUCUUCGAAGGUGUCACGUTT | ACGUGACACCUUCGAAGAATT |
